# Trends in Initial Hospitalizations of Patients with Newly Diagnosed Sjogren’s Disease in Poland Between 2012 and 2023: A Retrospective Data Analysis

**DOI:** 10.3390/jcm14061999

**Published:** 2025-03-15

**Authors:** Julia Domańska-Poboża, Łukasz Kapica, Krzysztof Kanecki, Katarzyna Lewtak, Paweł Goryński, Małgorzata Wisłowska

**Affiliations:** 1 Department of Rheumatology, National Institute of Geriatrics, Rheumatology and Rehabilitation, 02-637 Warsaw, Poland; 2 Department of Ergonomics, Central Institute for Labour Protection—National Research Institute, 00-701 Warsaw, Poland; 3 Department of Social Medicine and Public Health, Medical University of Warsaw, 02-005 Warsaw, Poland; 4 National Institute of Public Health NIH—National Research Institute, 00-791 Warsaw, Poland

**Keywords:** Sjögren’s disease, Sjögren’s syndrome, epidemiology, rheumatology, autoimmune disease

## Abstract

**Background/Objectives**: Sjögren’s disease (SjD) is a chronic autoimmune disease primarily affecting exocrine glands, often leading to systemic complications and comorbidities. While SjD is known to impact quality of life, research on hospitalization trends, demographic characteristics, and factors influencing hospital stay duration remains limited. This study aims to analyze hospitalizations due to SjD in Poland between 2012 and 2023, identifying key trends, risk factors, and healthcare implications. **Methods**: A retrospective analysis was conducted using data from the National General Hospital Morbidity Study, covering 13,999 first-time hospitalizations with an SjD diagnosis (ICD-10: M35.0). Descriptive statistics were applied to evaluate patient demographics, hospitalization trends, and comorbidities. The Mann–Whitney U test and chi-square test were used to compare groups, while a linear regression model identified predictors of hospital stay duration. **Results**: Women accounted for 90.3% of hospitalizations, with a median age of 57 years, compared to 53 years for men. The hospitalization rate fluctuated over time, with a decline in 2020, possibly due to the COVID-19 pandemic, followed by an increase in 2021–2023. The most common comorbidities included musculoskeletal disorders (17.8%), cardiovascular diseases (16.6%), and endocrine disorders (13.6%). Women had longer hospital stays than men (median 5 vs. 4 days, *p* < 0.001). Older patients and those admitted in emergency settings had significantly longer hospital stays. The overall mortality rate was low (0.2%), with a slightly higher but statistically insignificant mortality rate among men. **Conclusions**: The study highlighted the increasing burden of SjD-related hospitalizations and the need for improved outpatient management to reduce inpatient admissions. Factors such as older age, female sex, and emergency admissions were associated with prolonged hospitalization. Strengthening early diagnostic strategies, optimizing access to specialist care, and monitoring comorbidities could enhance patient outcomes and reduce hospital resource utilization.

## 1. Introduction

Sjögren’s disease (SjD, formerly referred to as Sjögren’s syndrome or Sjögren syndrome) is a chronic autoimmune disease primarily affecting exocrine glands, leading to sicca symptoms, such as xerophthalmia and xerostomia. However, beyond glandular dysfunction, SjD is a systemic condition with the potential to involve multiple organ systems, increasing the risk of severe complications, comorbidities, and long-term morbidity.

Although numerous studies have explored the immunological and clinical aspects of SjD, research focusing on hospitalization patterns, demographic characteristics, and factors influencing the length of hospital stay remains limited. Given the progressive nature of SjD and its frequent coexistence with other autoimmune and metabolic diseases, understanding these factors is crucial for optimizing healthcare strategies and improving patient outcomes.

This study aims to provide a comprehensive analysis of first-time hospitalizations associated with SjD in Poland between 2012 and 2023. By evaluating hospitalization frequency, patient demographics, comorbidities, and factors affecting the length of hospital stay, we seek to identify key trends that may inform better disease management, early intervention strategies, and healthcare resource allocation. Our findings can contribute to a more integrated approach to SjD care, emphasizing early diagnosis, outpatient management, and targeted interventions to reduce the burden of hospital admissions.

Sjögren’s disease is classified as either primary (pSjD) or secondary (sSjD), depending on whether it occurs independently or alongside other systemic autoimmune diseases [1,2]. The term “secondary” is most frequently used to describe SjD associated with rheumatoid arthritis (RA), systemic lupus erythematosus (SLE), or systemic sclerosis (SS). SjD can also coexist with other autoimmune conditions like celiac disease, autoimmune hepatitis, hypothyroidism, and Grave’s disease [3]. The ratio of pSjD to sSjD varies between sources: 1/3 in [4] and 2/5 in [5]. Studies indicate that SjD occurs in 19.5% of patients with rheumatoid arthritis and in 13.96% of patients with systemic lupus erythematosus, with female-to-male ratios of 14.7:1 and 16.82:1, respectively [6].

Sjögren’s disease primarily affects middle-aged women, with a female-to-male ratio of 9:1 [1,6,7]. The disease is most commonly diagnosed in the fifth or sixth decade of life [8]. The estimated prevalence of SjD is approximately 0.5%, with an annual incidence rate of 4 cases per 1000 individuals [2,9]. According to a meta-analysis by Qin et al. (2015), the cumulative incidence is 6.92 per 100,000 persons per year, while the prevalence is 60.82 cases per 100,000 inhabitants [8,9]. SjD can also be diagnosed in children, although this is less common [10].

Mortality analyses have shown that the leading causes of death in SjD patients are infections, cardiovascular diseases, and solid organ or hematologic malignancies. Although the overall mortality risk in SjD is not significantly higher than in the general population, it may be increased in patients with a more severe disease course. Factors contributing to a higher mortality risk include older age at diagnosis, male sex, parotid gland enlargement, extraglandular organ involvement, vasculitis, the presence of SS-B antibodies, hypocomplementemia, and cryoglobulinemia [11].

The pathogenesis of SjD is complex, involving genetic, environmental, and immune dysregulation factors. Genetic predispositions, such as specific HLA (human leukocyte antigen) alleles and polymorphisms in IFN (interferon) pathways, along with epigenetic modifications, contribute to disease susceptibility [12]. Environmental factors, like viral infections (e.g., Epstein–Barr virus), may trigger SjD through molecular mimicry and apoptosis [13]. Immune dysregulation occurs with epithelial cells actively presenting antigens and secreting proinflammatory cytokines, while overactivation of the IFN pathway stimulates B-cells and autoantibody production. T-cells (Th1 and Th17) further amplify inflammation [14]. The hallmark histological feature is focal lymphocytic sialadenitis (FLS), leading to glandular damage and systemic complications, including dry eyes, mouth, and potential involvement of the lungs, kidneys, and nervous system [15]. Severe cases may lead to non-Hodgkin lymphoma [16].

Sjögren’s disease manifests through a variety of glandular and systemic symptoms. The most common signs are dryness of the eyes and mouth, affecting nearly all patients. This dryness can cause difficulty swallowing (dysphagia), altered taste (dysgeusia), and discomfort, such as pain and burning sensations. Dryness of the oral mucosa, dental issues like caries, and enlarged salivary glands, particularly the parotid glands, are also frequently observed. Ocular symptoms, including dry eyes, photosensitivity, and foreign body sensations, are common, often exacerbated in dry or windy environments, potentially causing long-term corneal damage. Other glandular manifestations include dryness of the respiratory tract, leading to hoarseness and a dry cough, as well as reduced vaginal secretion and gastrointestinal dysfunction [16,17,18]. Systemically, fatigue, sleep disorders, anxiety, and chronic pain are prevalent in 70–80% of patients. Musculoskeletal symptoms such as joint pain, muscle pain, and morning stiffness are common, though arthritis is less frequent [19]. Dermatologically, patients may experience dry skin (xerosis), annular erythema, and vasculitic lesions [20]. In up to 20% of cases, pulmonary issues such as dry cough, bronchiolitis, or interstitial lung disease (ILD) are seen, while cardiac involvement is rarer, though it can include cerebrovascular events and myocardial infarction [21,22]. Neurological involvement may include neuropathy, cognitive dysfunction, and sleep disturbances [23]. Renal and gastrointestinal complications like chronic nephritis and motility disorders are also common [24,25]. Furthermore, SjD carries an increased risk of non-Hodgkin lymphoma, particularly in active disease areas like the salivary glands [1]. In pregnancy, women with SjD face an elevated risk of miscarriage, preterm birth, and neonatal lupus, with some babies experiencing congenital heart block [26].

Sjögren’s disease is diagnosed using the 2016 ACR/EULAR (American College of Rheumatology/European Alliance of Associations for Rheumatology) classification criteria, which combine elements of previous systems to improve patient selection for clinical trials. These criteria focus on primary SjD and require a score of ≥4 based on objective markers, such as anti-SSA/Ro (anti-Sjögren’s syndrome-related antigen A) antibody positivity, ocular staining scores, Schirmer’s test results, and salivary gland function [27].

Autoantibodies, including ANA (antinuclear antibodies), RF (rheumatoid factor), anti-Ro/SSA, and anti-La/SSB (anti-Sjögren’s syndrome-related antigen B), are key immunological markers for SjD, though isolated anti-La/SSB positivity is excluded [27,28]. Labial salivary gland biopsy remains crucial, especially in seronegative patients, as it confirms lymphocytic infiltration and assesses lymphoma risk [15,29]. Other diagnostic tests include Schirmer’s test for tear production, ocular staining to evaluate eye surface damage, and sialometry to measure unstimulated saliva flow [27]. SjD diagnosis requires comprehensive clinical evaluation to rule out other conditions. Classification criteria help guide diagnosis but should be used alongside clinical judgment.

To assess disease severity, EULAR developed two indices: ESSPRI (EULAR Sjögren’s Syndrome Patient Reported Index) and ESSDAI (EULAR Sjögren’s Syndrome Disease Activity Index). A newer version, ClinESSDAI, omits immunological parameters to better assess clinical manifestations. These tools help monitor disease progression and evaluate treatment effectiveness [30,31].

The treatment of Sjögren’s disease requires an interdisciplinary approach, encompassing both symptomatic therapy for sicca symptoms and immunosuppressive treatment in cases of organ involvement. Due to the lack of a curative method, treatment focuses on symptom relief and improving patients’ quality of life, necessitating collaboration among various specialists, including general practitioners, immunologists, rheumatologists, ophthalmologists, otolaryngologists, and dentists.

For the management of dry mouth (xerostomia), fluoride, artificial saliva, and saliva-stimulating medications, such as pilocarpine and cevimeline, are used. Patients are advised to avoid factors that exacerbate dryness, such as xerogenic medications, caffeine, alcohol, and smoking. For dry eyes (xerophthalmia), tear substitutes, eye drops, ointments, and, in more severe cases, cyclosporine are employed. In some instances, procedures such as punctal occlusion or corneal transplantation may be necessary. The treatment of dry skin (xeroderma) and nasal dryness involves the use of emollients and moisturizing agents [32,33].

Systemic symptoms, such as fatigue and arthritis, are managed with anti-inflammatory medications, including hydroxychloroquine, methotrexate, azathioprine, and mycophenolate. In more advanced cases, when symptoms are refractory to conventional treatment, immunosuppressive drugs such as rituximab, cyclophosphamide, or belimumab are used. Fatigue management may also involve regular physical exercise [32,33].

The use of immunosuppressive drugs in the treatment of Sjögren’s disease, particularly in cases with systemic involvement, carries a risk of infectious complications, including opportunistic infections and viral reactivations [34]. Prolonged therapy may also lead to hematologic abnormalities, organ toxicity, metabolic disturbances, and bone loss. Regular monitoring and careful risk assessment are essential to minimize these complications [35,36,37,38].

## 2. Materials and Methods

### 2.1. Data Sources

This retrospective study aimed to characterize selected factors related to the hospitalization of patients with a first-time diagnosis of Sjögren’s syndrome over the years 2012–2023. The study was based on anonymized hospital morbidity data obtained from the National General Hospital Morbidity Study, conducted by the National Institute of Public Health NIH—National Research Institute.

The dataset included anonymized patient records. The data were systematically reported by hospitals using the General Hospital Statistical Card (Mz/Szp-11), ensuring high completeness and accuracy due to the legal obligation for inpatient healthcare facilities to report hospitalizations.

### 2.2. Study Population

The study population consisted of all hospitalizations in Poland between 1 January 2012 and 31 December 2023, during which the ICD-10 code M35.0 (Sjögren’s disease) appeared for the first time. The inclusion criteria for this analysis were:

1. The presence of ICD-10 code M35.0 as either the principal or additional diagnosis.
2. Hospital admission recorded within the study period (2012–2023).
3. The ICD-10 code M35.0 appearing for the first time during the hospitalization.

Hospitalizations that did not meet these criteria were excluded from the study. Based on these criteria, and with the caveat of potential coding inaccuracies, the study can be considered to include all initial hospitalizations of patients with a newly diagnosed Sjögren’s disease. The final dataset comprised 13,999 hospitalization records, with a female predominance (12,642 cases; 90.3%) and a smaller proportion of male patients (1357 cases; 9.7%). This study covered nearly 100% of hospitalizations for SjD in Poland, ensuring a robust and representative sample for epidemiological assessment.

### 2.3. Statistical Methodology

The statistical analysis comprised descriptive statistics, including frequency distributions, as well as the calculation of mean and median for quantitative variables. The Mann–Whitney U test and the chi-square test were applied. A significance threshold of *p* < 0.05 was established, and given the large sample size, effect size measures were also reported. Additionally, a linear regression model was employed to analyze the data. Predictor multicollinearity was evaluated using the variance inflation factor (VIF). Variables were introduced into the model in sequential blocks to assess changes in the R-squared value with each added predictor. All statistical analyses were performed using the R software package (version 4.3.3).

### 2.4. Use of GenAI in Writing

During the preparation of this work, the authors used ChatGPT (Model GPT-4o) for language correction. After using these tools/services, the authors reviewed and edited the content as needed and therefore take full responsibility for the content of the publication.

## 3. Results

### 3.1. General Findings

A total of 13,999 hospitalizations related to Sjögren’s disease were analyzed. The majority of hospitalized patients were women (90.3%; n = 12,642), while men accounted for 9.7% (n = 1357). The age of patients ranged from 1 to 95 years, with a median age of 56 years (IQR = 22). The data suggest that Sjögren’s disease is primarily diagnosed in middle-aged and older adults, with a peak prevalence observed in patients aged 50–60 years.

### 3.2. Trends in Hospitalization

**Figure 1:**
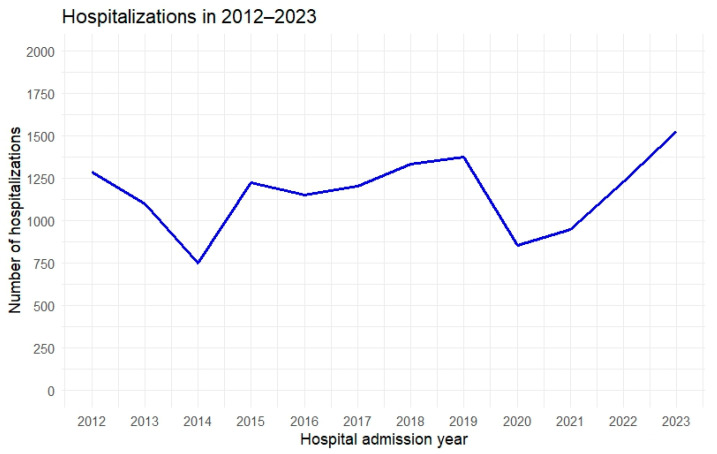
Number of hospitalizations by year.

Between 2012 and 2023, the hospitalization rate for patients with Sjögren’s disease showed significant fluctuations. Figure 1 presents the number of hospitalizations in the respective years covered by the analysis. From 2012 to 2014, hospitalizations declined, reaching their lowest point in 2014, likely due to improved disease management, changes in hospitalization criteria, or healthcare system adjustments. However, from 2015 onward, hospitalizations increased, possibly reflecting a higher diagnostic rate or changes in treatment and admission practices.

A period of relative stability between 2016 and 2019 suggests the healthcare system adapted to these trends. In 2020, a sharp decline occurred, likely due to the COVID-19 pandemic limiting hospital access for non-COVID cases. From 2021, a steady increase in hospitalizations was observed, potentially due to delayed admissions from the pandemic and the return to normal hospital operations. By 2023, hospitalizations reached their highest level in the analyzed period, possibly indicating a growing need for hospital treatment in Sjögren’s disease or increased diagnostic awareness among physicians.

### 3.3. Demographic Characteristics

**Figure 2:**
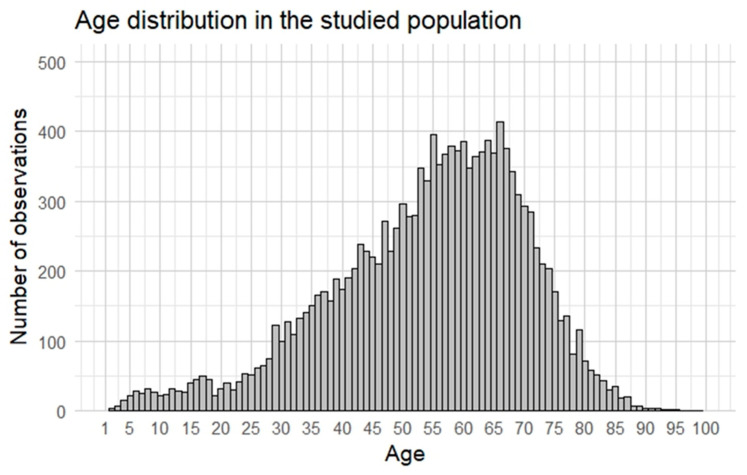
Age distribution of the patients.

The patients’ ages ranged from 1 to 95 years, with a median age of 56 years (IQR: 22). Females had a slightly higher median age (57 years) compared to males (53 years), and this difference was statistically significant (*p* < 0.001). The mean age for all participants was 54.2 years, with females having a similar mean age (54.8 years) and males being significantly younger (48.8 years). These findings align with the well-documented higher prevalence of Sjögren’s disease among middle-aged and older women. Figure 2 shows the age distribution of the patients.

There were significantly more rural inhabitants than urban dwellers (N = 4439, 31.7% vs. N = 9507, 67.9%; *p* < 0.001; 53 missing data points regarding the size of the place of residence were recorded). The distribution of residence also differed between genders, with a slightly higher proportion of men living in urban areas (36.0%) compared to women (31.3%) (*p* < 0.001). This suggests a potential disparity in healthcare access or disease recognition between urban and rural populations. Table 1 provides an overview of demographic characteristics.

### 3.4. Length of Hospital Stay and Mortality

The length of stay varied widely, ranging from 0 to 373 days, with a median of 5 days for all participants. Males had a shorter median hospital stay (4 days) compared to females (5 days), and this difference was statistically significant (*p* < 0.001). The mean hospital stay was also slightly longer for females (6.48 days) than for males (5.71 days), suggesting that women with Sjögren’s disease might require more extended inpatient care.

The overall mortality rate among hospitalized patients was very low (0.2%, with 28 recorded deaths). Mortality was slightly higher in males (0.3%) compared to females (0.1%), but the difference was not statistically significant (*p* = 0.411). This indicates that while Sjögren’s disease itself is not directly associated with high mortality, comorbidities and complications might contribute to rare cases of fatal outcomes.

### 3.5. Admission Type and Hospital Departments

The majority of hospitalizations were planned admissions (80.7%; n = 11,294; among females: 80.7%; n = 10,203; among males: 80.4%; n = 1901), while emergency admissions constituted 19.2% (n = 2686) (*p* < 0.001). Most patients were admitted to the department of rheumatology (69.7%), followed by the department of internal medicine (7.9%). This distribution suggests that SS patients are primarily managed in specialized hospital units rather than general medical wards.

### 3.6. Comorbid Conditions

A total of 9837 patients (70.3%; among females: 70.1%; n = 8856; among males: 72.4%; n = 981) had the ICD-10 code M35.0 as their primary diagnosis, while the remaining patients had M35.0 as a comorbid condition. Table 2 outlines the most common comorbidities among hospitalized patients. The most prevalent coexisting conditions are listed below.

- Diseases of the musculoskeletal system and connective tissue (M00–M99) were the most frequent comorbid conditions, affecting 17.8% of hospitalizations. This aligns with the known autoimmune nature of Sjögren’s disease, which frequently coexists with other rheumatologic conditions. When stratified by sex, musculoskeletal and connective tissue diseases were similarly common in both females (17.9%) and males (16.9%).
- Diseases of the circulatory system (I00–I99) were the second most common comorbidity with Sjögren’s disease, which has been previously reported in autoimmune diseases. Circulatory system diseases affected 16.8% of females and 15.3% of males, indicating a slightly higher cardiovascular burden among women.
- Endocrine, nutritional, and metabolic diseases (E00–E90) accounted for 13.6% of hospitalizations, indicating a possible association with metabolic disorders such as diabetes or thyroid dysfunction, which are known to co-occur with autoimmune diseases. These conditions were more prevalent among females (14.2%) compared to males (8.7%). Notably, a significant proportion of patients also had autoimmune comorbidities, including rheumatoid arthritis and systemic lupus erythematosus, which may indicate a broader spectrum of systemic autoimmune involvement.

The low proportion of infection-related codes among patients hospitalized for the first time with a diagnosis of Sjögren’s disease may stem from the absence of immunosuppressive treatment at the time of diagnosis, which would otherwise increase susceptibility to infections.

### 3.7. Predictors of Length of Stay (LOS)

Table 3 presents the results of the linear regression analysis predicting LOS in the analyzed group. The results presented in the table indicate that women had a significantly longer LOS (M = 6.48; Me = 5.00) than men (M = 5.71; Me = 4.00), although the relationship was not strong. It was also found that the older the patient, the longer the LOS. Rural residents had a longer LOS than urban residents (M = 6.70; Me = 6 vs. M = 6.28; Me = 5). An important predictor of LOS was also the emergency mode of admission (M = 8.57; Me = 8 compared to M = 5.86; Me = 5 for the planned mode of admission).

## 4. Discussion

This study analyzed hospitalization data for patients diagnosed with Sjögren’s disease in Poland between 2012 and 2023, providing a comprehensive overview of hospitalization frequency, demographic characteristics, and clinical factors influencing the length of hospital stay.

The analysis of 13,999 hospitalizations revealed a significant predominance of women (90.3%), which aligns with the commonly reported female-to-male ratio in SjD (9:1) [1,6,7]. The median age of female patients was 57 years, while for male patients, it was 53 years, consistent with epidemiological data indicating the most frequent diagnosis occurring in the fifth or sixth decade of life [8]. However, it is noteworthy that the median age among hospitalized men was slightly lower, which may suggest either an earlier onset of symptoms requiring hospitalization or a different disease progression pattern in men.

Demographic differences between rural and urban patients, including longer hospital stays among rural residents, may indicate disparities in access to medical care, delayed specialist consultations, or a different disease burden profile in the rural population.

During the study period, fluctuations in the number of hospitalizations were observed: a decline from 2012 to 2014, followed by an increase starting in 2015, a period of relative stabilization between 2016 and 2019, and then a sharp drop in 2020, likely related to the COVID-19 pandemic. There are reports, based on an analysis of 53 articles published between December 2019 and September 2021, indicating that the COVID-19 pandemic and the measures adopted significantly affected access to healthcare for patients with non-COVID-19 conditions. A generalized reduction in the use of health services was observed, particularly in the early stages of the pandemic. The most frequently identified barriers to accessing care for non-COVID-19 patients were related to both the healthcare system and the population. On the system side, the primary issue was a lack of resources, while on the population side, predisposing factors, such as fear of contagion, stigma, and anticipation of access difficulties, were common. Enabling factors, including lower socioeconomic status and increased technological barriers, further limited access. Overall, the pandemic not only introduced new obstacles but also exacerbated pre-existing inequalities in access to care [39,40].

The increases observed after 2021 may indicate a “catch-up” effect in diagnostic and therapeutic backlogs and a return to the standard hospital admission process. Additionally, 2023 recorded the highest hospitalization rate, which may reflect both improved disease detection (increased diagnostic vigilance among physicians of various specialties) and a genuine rise in the demand for hospital treatment due to the development of complications and comorbidities.

Women remained in the hospital longer than men (median 5 vs. 4 days), which may be related to differences in disease progression, a higher prevalence of coexisting autoimmune disorders among female patients [1,2], or differing therapeutic needs. A correlation was also observed between age and hospitalization duration: older patients required longer hospital stays, potentially due to more frequent complications, a greater number of comorbidities, or the need for more complex diagnostics [11,41,42]. Similarly, emergency admissions were significantly associated with longer hospital treatment—patients admitted with acute deterioration, symptom exacerbation, or with complications typically require comprehensive care and in-depth diagnostics.

According to previous reports, the low mortality rate in the studied group (0.2%) suggests that Sjögren’s disease itself is not, in most cases, a direct cause of high mortality [11]. However, the slightly higher mortality among men (though statistically insignificant) may indicate a more severe course of the disease in this group, warranting further research into immunological mechanisms and hormonal differences.

Additionally, pSjD is a chronic, slowly progressing disease that does not pose a direct life threat to most patients, as evidenced by a 10-year cumulative survival rate exceeding 90%. Subgroup analyses and sensitivity tests have not demonstrated a significant increase in mortality among pSjD patients.

The slightly higher mortality among men (though statistically insignificant) may suggest a more severe course of the disease in this group, requiring further research into immunological mechanisms and hormonal differences.

The most common coexisting conditions were musculoskeletal disorders (17.8%), cardiovascular diseases (16.6%), and endocrine disorders (13.6%). This can be attributed, on one hand, to the frequent coexistence of SjD with other rheumatic diseases, such as rheumatoid arthritis and systemic lupus erythematosus [2,6], and on the other hand, to the widespread prevalence of cardiovascular and metabolic diseases, particularly among older individuals. The presence of various coexisting endocrine disorders (e.g., thyroid diseases) is also consistent with previous observations that patients with autoimmune diseases are at higher risk of developing multi-organ autoimmune disorders [1,2].

The study results confirmed that SjD poses a significant challenge to the healthcare system, as evidenced by the increasing number of hospitalizations in recent years. The obtained data suggest the need for:

- Strengthening outpatient care and early diagnosis, particularly in high-risk populations (middle-aged women and patients suspected of having other autoimmune diseases), which could help reduce the necessity for planned hospitalizations.
- Improving access to specialists (rheumatologists, ophthalmologists, dentists, and endocrinologists) in outpatient care, especially in smaller towns, to prevent exacerbations and complications requiring hospital treatment.
- Addressing the mode of patient admissions—emergency hospitalizations are associated with a significant increase in hospital stay duration, indicating that optimizing the diagnostic and therapeutic process in outpatient consultations could reduce the number of acute admissions.
- Monitoring coexisting diseases, particularly endocrine and cardiovascular conditions, which may improve patient prognosis and quality of life.

The study has several limitations that should be taken into account when interpreting the results. Most of these limitations stem from the nature of the data source, which relied on administrative hospital discharge records containing only ICD-10 diagnostic codes, along with basic demographic and hospitalization information. These records do not provide a complete clinical picture of patients, as they lack detailed medical histories, outpatient care data, and follow-up information. Consequently, it was not possible to track patient outcomes after discharge, preventing the assessment of long-term disease progression, readmission rates, or treatment effectiveness. Furthermore, the dataset did not include clinical severity indicators, laboratory results, or standardized disease activity measures such as ESSDAI or ESSPRI, precluding the stratification of patients by disease activity and limiting insights into the relationship between disease severity and hospitalization. The absence of such data restricts the ability to fully assess the clinical conditions of hospitalized patients and their longer-term outcomes. Additionally, the influence of specific pharmacological treatments, such as hydroxychloroquine, rituximab, or other immunosuppressive therapies, on hospitalization rates could not be assessed due to the lack of detailed treatment data within the administrative records. Therefore, while the study offers valuable insights into the epidemiology and hospitalization patterns of Sjögren’s disease in Poland, it does not capture the full clinical spectrum, long-term burden, or post-hospitalization disease course. Future research based on clinical registries or prospective cohort studies, incorporating detailed clinical data and follow-up information, will be essential to better understand the long-term outcomes, the specific treatment effectiveness, and the impact of disease severity on hospitalization patterns and overall patient prognosis.

Another important limitation is the reliance on ICD-10 codes for identifying cases of Sjögren’s disease. Although the ICD-10 system is a widely used classification tool, its accuracy depends on correct coding practices by healthcare providers, which may be influenced by differences in clinical experience, institutional policies, and coding conventions. Misclassification, undercoding, or overcoding of Sjögren’s disease cannot be entirely excluded and may have impacted the completeness and accuracy of case identification. This inherent limitation should be considered when interpreting the findings and highlights the need for future studies using clinical registries or validated diagnostic criteria applied directly at the point of care.

Furthermore, retrospective studies are subject to confounding factors that cannot always be accounted for, such as differences in hospital admission criteria, regional disparities in healthcare access, and variations in clinical management strategies over time. Additionally, missing values in the dataset necessitated exclusions, which may introduce selection bias. These factors may influence hospitalization trends and patient outcomes.

Despite these limitations, our study provides valuable epidemiological insights due to the large sample size and the nationwide coverage of the database, which captures nearly 100% of hospitalizations in Poland. This extensive dataset enhances the reliability of our findings and allows for meaningful observations on hospitalization trends in Sjögren’s disease. Future prospective studies incorporating clinical, biochemical, and pharmacological data would help address the limitations of this analysis and provide a more comprehensive understanding of the disease course and treatment outcomes.

## 5. Conclusions

The conducted study confirms that Sjögren’s disease is most frequently diagnosed in middle-aged and older women, with them being the most frequently hospitalized, and the dynamics of hospitalization between 2012 and 2023 reflect both changes in the availability of hospital services and increasing clinical vigilance. Longer hospitalizations in women, older individuals, and in emergency settings highlight the importance of early diagnosis and effective conservative treatment, which can reduce the number of flare-ups requiring hospital intervention. The low mortality rate in the analyzed group suggests that although SjD often causes a significant decrease in quality of life and requires frequent healthcare interactions, it rarely directly leads to death. However, the growing awareness of the role of comorbidities (endocrine, cardiological, and others) in the course of SjD emphasizes the need for further research and the development of integrated care models for patients.

## Figures and Tables

**Table 1:** Demographic and clinical data for the comparison of male and female groups.

Variable		All Participants	Females	Males	*p*	Effect Size
Age					<0.001 ^a^	0.150 ^c^
	Min–Max	1–95	1–95	2–90		
	Me (IQR)	56 (22)	57 (21)	53 (28)		
	M (SD)	54.2 (15.9)	54.8 (15.3)	48.8 (19.8)		
Place of residence					<0.001 ^b^	0.031 ^d^
City	N (%)	4439 (31.8%)	3951 (31.3%)	488 (36.0%)		
Village	N (%)	9507 (68.2%)	8659 (68.5%)	858 (63.4%)		
Lengths of stay (LOS)					<0.001 ^a^	0.097 ^c^
	Min–Max	0–373	0–373	0–53		
	Me (IQR)	5 (6)	5 (6)	4 (6)		
	M (SD)	6.40 (6.85)	6.48 (6.97)	5.71 (5.55)		
Number of deaths					0.411 ^b^	0.007 ^d^
	N (%)	28 (0.2%)	24 (0.1%)	4 (0.3%)		

^a^ Mann–Whitney U test; ^b^ chi-square test; ^c^ Glass’s rank correlation coefficient; ^d^ Cramér’s V coefficient.

**Table 2:** The frequencies of comorbid conditions in the analyzed period.

	N (Percentage of Hospitalizations)		
ICD-10 Code	All Participants	Females	Males
Certain infectious and parasitic diseases A00–B99	141 (1.0%)	115 (0.9%)	26 (1.9%)
Neoplasms C00–D49	353 (2.5%)	311 (2.5%)	42 (3.1%)
Diseases of the blood and blood-forming organs and certain disorders involving the immune mechanism D50–D89	422 (3.0%)	391 (3.1%)	31 (2.3%)
Endocrine, nutritional, and metabolic diseases E00–E90	1907 (13.6%)	1789 (14.2%)	118 (8.7%)
Mental and behavioral disorders F00–F99	105 (0.7%)	99 (0.8%)	6 (0.4%)
Diseases of the nervous system G00–G99	324 (2.3%)	297 (2.4%)	27 (2.0%)
Diseases of the eye and its adnexa, ear, and mastoid process H00–H95	205 (1.5%)	189 (1.5%)	16 (1.2%)
Diseases of the circulatory system I00–I99	2328 (16.6%)	2120 (16.8%)	208 (15.3%)
Diseases of the respiratory system J00–J99	773 (5.5%)	657 (5.2%)	116 (8.5%)
Diseases of the digestive system K00–K93	770 (5.5%)	686 (5.4%)	84 (6.2%)
Diseases of the skin and subcutaneous tissue L00–L99	326 (2.3%)	295 (2.3%)	31 (2.3%)
Diseases of the musculoskeletal system and connective tissue other than ankylosing spondylitis M00–M99 without M35	2494 (17.8%)	2265 (17.9%)	229 (16.9%)
Diseases of the genitourinary system N00–N99	560 (4.0%)	493 (3.9%)	67 (4.9%)
Pregnancy, childbirth, and the puerperium O00–O99	65 (0.5%)	65 (0.5%)	0
Symptoms, signs, and abnormal clinical and laboratory findings, not elsewhere classified R00–R99	248 (1.8%)	220 (1.7%)	28 (2.0%)
Injury, poisoning, and certain other consequences of external cause S00–T98	57 (0.4%)	49 (3.9%)	8 (0.6%)

**Table 3:** Results of the linear regression analysis predicting LOS.

Predictor	B (SE)	β	*p*	R^2^_adj_.	Model Comparison	
					ΔR^2^	*p*
Intercept	2.53 (0.23)		<0.001			
Gender (0—Males, 1—Females)	0.39 (0.17)	0.02	0.023	0.002		
Age	0.04 (0.003)	0.17	<0.001	0.03	0.03	<0.001
Size of the place of residence (0—village, 1—city)	−0.66 (0.11)	−0.05	<0.001	0.03	0.003	<0.001
Mode of admission (0—planned, 1—emergency)	2.57 (0.13)	0.17	<0.001	0.06	0.03	<0.001

B—unstandardized coefficient; SE—standard error; β—standardized coefficient.

## Data Availability

Data are available upon reasonable request according to ICMJE requirements. Data shared: All the individual participant data were collected during the trial, after deidentification. Available documents: study protocol, statistical analysis, analytic code. When will data be available: beginning three months and ending two years following the article’s publication. With whom: researchers who provide a methodologically sound proposal. For what type of analyses: to achieve the aim of the approved proposal. By what mechanism will data be made available: The proposal should be directed to julia-domanska03@wp.pl.
